# Texture profile analysis reveals a stiffer ovarian cortex after testosterone therapy: a pilot study

**DOI:** 10.1007/s10815-019-01513-x

**Published:** 2019-07-20

**Authors:** C. De Roo, K. Tilleman, C. Vercruysse, H. Declercq, G. T’Sjoen, S. Weyers, P. De Sutter

**Affiliations:** 1 Department of Reproductive Medicine, Ghent-Fertility and Stem Cell Team (G-FaST), Ghent University Hospital, 9000 Ghent, Belgium; 2 Bio print Core Facility, Tissue Engineering and Biomaterials, Department of Basic Medical Science, Faculty of Medicine and Health Science, Ghent University, 9000 Ghent, Belgium; 3 Department of Endocrinology and Center for Sexology and Gender, Ghent University Hospital, 9000 Ghent, Belgium

**Keywords:** Cortex, Ovary, Stroma, Testosterone, Transgender, Texture profile analysis

## Abstract

**Purpose:**

The importance of the surrounding ovarian stromal cells and extracellular matrix in the development and maturation of follicles has recently gained attention. An aberrant extracellular matrix has been described in ovaries of patients with polycystic ovary syndrome where a more rigid structural environment, possibly induced by endogenous testosterone, impairs normal folliculogenesis. In this context, we describe the textural parameters of the ovarian cortex of transgender men after prolonged testosterone administration compared to the textural parameters of the non-exposed ovarian cortex originating from female oncological patients.

**Methods:**

Texture profile analysis (TPA) was performed on ovarian cortex (5 × 5 mm) of oncological and transgender patients in order to measure stiffness, hardness, cohesiveness, and springiness of the ovarian cortex (LRXplus universal testing system). Statistical analysis was performed using repeated measurements mixed models and the Spearman rank order correlation test (IBM SPSS Statistics 23).

**Results:**

A total of 36 frozen-thawed cortical strips (5 × 5 mm) were subjected to TPA. The superficial part of cortex fragments originating from transgender persons (fragments < 1.4 mm; *N* = 10) appeared to be significantly stiffer compared to cortex derived from oncology patients (fragments < 1.4 mm; *N* = 7) (6.78 ± 1.38 N/mm versus 5.41 ± 0.9 N/mm respectively, *p* = 0.036).

**Conclusions:**

This is the first application of TPA in ovarian cortex to study the physical properties. Comparing the physical properties, we objectively describe an increased cortical stiffness in the most outer part of the ovarian cortex following prolonged testosterone administration in transgender men compared to the ovarian cortex of oncological patients. This preliminary and novel approach could be the start of future research to understand the physical properties of ovarian tissue.

**Electronic supplementary material:**

The online version of this article (10.1007/s10815-019-01513-x) contains supplementary material, which is available to authorized users.

## Introduction

The importance of the surrounding ovarian stromal cells and extracellular matrix in the development and maturation of follicles has recently gained attention [1]. The extracellular matrix does not only contribute to the structural support of the growing follicle but also to the bidirectional paracrine communication between the oocyte and surrounding granulosa and theca cells thereby influencing folliculogenesis [1, 2]. Also, the role of tissue pressure has been demonstrated in clinical studies in ovarian cortex transplantation [3]. An aberrant extracellular matrix has been described in ovaries of patients with polycystic ovary syndrome (PCOS), resulting in a more rigid structural environment for the residing follicles and causing a dysfunctional follicle development [4]. Both theca cell hyperplasia *in vivo* and a more rigid alginate artificial ovary matrix *in vitro* have been correlated with an increased androgen production [5], illustrating a possible effect of mechanotransduction (the conversion of mechanical stimuli into biological responses) on ovarian hormone synthesis. This androgen production is potentially triggering a vicious circle, as androgen receptor expression is described in stromal cells [6–8] and administration of testosterone in transgender men (female to male transgender persons) is known to cause a thicker cortex with ovarian stromal hyperplasia [9]. Although a previous study of our group revealed no quantitative difference in follicle counts and distributions in this patient group, testosterone could influence the surrounding matrix [10] and in turn have an effect on the development of the follicles when the stored tissue is used for further purposes. While manipulating ovarian tissue, a difference in rigidness was noticed in comparison to manipulation of ovarian cortex originating from oncological patients. To quantify and analyze this observation, a texture profile analysis (TPA) was performed.

TPA is a double compression test, developed for determining the textural properties of foods, but also used for defining the possible application of other matrices such as gels, pharmaceuticals, and biomaterial research [11, 12]. By twice compressing the target of interest, different textural parameters can be defined in a single experiment, such as stiffness, hardness, cohesiveness, and springiness [12]. In this work, we describe the textural parameters of the ovarian cortex of transgender men compared to the textural parameters of the cortex originating from oncological female patients.

## Materials and methods

### Study design and ethical approval

Ethical approval was obtained from the Ghent University Hospital institutional review board under reference 2012/780, with Belgian registration number B670201 21 5468. A written informed consent was obtained from all patients. In total, 36 ovarian cortex pieces of in total 6 patients were used in this study. From these 6 patients, ovarian tissue was obtained of 3 deceased oncology patients (25.00 ± 3.61 years), who consented to donate their cryopreserved ovarian tissue to research postmortem. They underwent a unilateral oophorectomy for fertility preservation purposes prior to chemotherapy. Oncological indications for fertility preservation comprised acute lymphoblastic lymphoma, breast cancer, and acute myeloid leukemia. None of them received chemo and/or radiotherapy before the oophorectomy. In parallel, 3 age-matched transgender men (22.67 ± 2.08 years) consented to donate their ovarian tissue upon bilateral oophorectomy for gender confirming surgery. They all received intramuscular testosterone undecanoate 1000 mg every 12 weeks during 80.72 ± 32.24 (56.43, 68.43, and 117.29 respectively) weeks as cross-sex hormone treatment prior to the oophorectomy. In the oncological cohort, per patient, 4 cortical fragments of 5 × 5 mm^2^ were analyzed per patient. In the transgender group, we were able to include 8 cortical fragments of 5 × 5 mm^2^ per patient.

### Ovarian tissue collection and processing

Immediately after hysterectomy with bilateral oophorectomy, the entire ovaries were transported to the laboratory on ice, in Leibovitz L-15* medium (Life Technologies, Ghent, Belgium), supplemented with 0.45% human serum albumin (Red Cross, Belgium). The ovaries were bisected, and the medulla was carefully removed by scraping with a scalpel to prepare the cortical tissue. This cortex was subsequently fragmented in pieces of 5 × 5 mm^2^. The fragments were cryopreserved by controlled slow-freezing and stored in liquid nitrogen as previously described [10].

Prior to the texture profile analysis, the vial containing the cortical fragment was thawed in a warm water bath at 37 °C for 2 min. Following 3 wash steps in Leibovitz L-15* medium (Life Technologies, Ghent, Belgium), supplemented with 0.45% human serum albumin (Red Cross, Belgium) for 5 min, an exact cortical surface of 5 × 5 mm^2^ was obtained by cutting with a scalpel and checking all edges with a ruler.

### Histology

Ovarian cortex was fixed in 10% paraformaldehyde for 4 h at 4 °C, embedded in paraffin, and serially sectioned at 5 μm, perpendicular to the ovarian surface. Sections were stained with (Mayer) hematoxylin (Merck, Overijse, Belgium) and eosin (Thermo Scientific, Merelbeke, Belgium) for histologic analysis. Sections were analyzed using an inverted microscope with a × 20 and × 40 magnification.

### Texture profile analysis or compression analysis

**Fig. 1:**
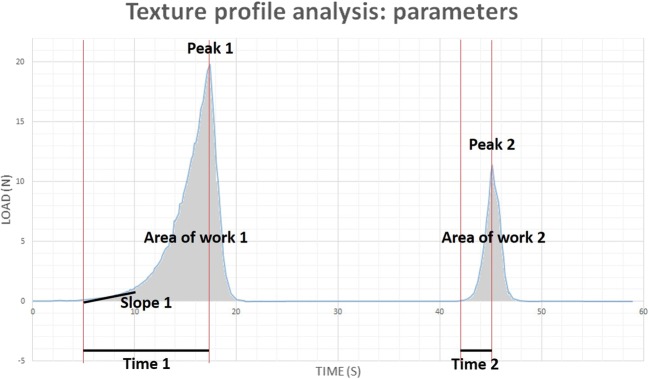
Texture profile analysis parameters. Stiffness = slope of the toe region (0.1–1 N) of the curve (slope 1). Hardness 1 and 2 = peak force during the first and second cycle respectively (peaks 1 and 2). Cohesiveness = the ratio of the work of the second cycle to that of the first cycle (area of work 2 divided by area of work 1). Springiness = the division of the distance of the detected height on the second cycle by the original compression time (Fig. 1: time 2 divided by time 1)


Cortical strips (5 × 5 mm) were compressed at room temperature over a distance of 50% of the original sample thickness during 2 cycles (S1 video). A universal testing machine (LRXplus, Lloyd Instruments) was used for compression at a rate of 5 mm/min. Stiffness was determined during the first cycle as the slope of the toe region (0.1–1 N) of the curve (Fig. 1: slope 1). Hardness values were recorded as the peak force during the first and second cycle (Fig 1: peaks 1 and 2 respectively). Cohesiveness (a measure for withstanding compression) was calculated by the ratio of the work of the second cycle to that of the first cycle (Fig. 1: area of work 2 divided by area of work 1). Springiness (a measure for elasticity) is the rate at which deformation is re-established and was determined as the division of the time of the detected height on the second cycle by the original compression time (Fig. 1: time 2 divided by time 1).

ESM 1S1 Video texture profile analysis of ovarian cortex. (MP4 60466 kb)


### Statistical analysis

Statistical analysis was conducted with IBM SPSS Statistics 23 (IBM Corp., New York, USA). TPA parameters were analyzed using repeated measurements mixed models. Correlation analysis was conducted through inspection of scatter plots to rule out other correlations than linear and by using Spearman’s rank-order correlation tests. *p* < 0.05 was considered to be statistically significant.

## Results

### Textural parameters of ovarian cortex

**Fig. 2:**
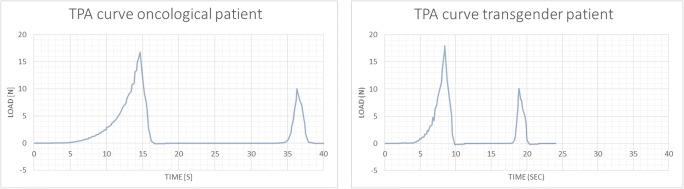
Representative texture profile analysis curves of ovarian cortex derived from oncological patients and transgender men. S seconds, N Newton

In total, 36 cortex fragments were processed for TPA (S1 video), of which 2 analyses (1 fragment of a transgender person and 1 fragment from an oncological patient) were excluded from the results due to technical errors (1 exclusion because of wrong TPA settings and 1 because of an error during compression). A typical TPA double compression curve of ovarian cortex is characterized by a slower upstroke in the toe (0.1–1 N) region, where increasing compression resulted in a steeper upstroke prior to the peak. Release of the maximum compression causes an immediate drop in the TPA curve. Typically, a bigger first curve was noted, when compared to the second curve (peak 2 < peak 1), reflecting an incomplete recovery of the tissue postcompression, probably as a result from the breaking points seen in the first upstroke (Fig. 2).

### Comparison between textural profiles of cortex originating from transgender and oncological patients

There was no difference in thickness of the cortex fragments when comparing the 2 groups with a mean of 1.43 ± 0.46 mm in the transgender cohort compared to 1.38 ± 0.38 mm in the oncological group (*p* = 0.826). For this comparable thickness, no difference in texture stiffness could be observed (transgender stiffness 5.20 ± 1.90 N/mm compared to oncological stiffness 5.11 ± 1.15 N/mm, *p* = 0.970). There was no significant difference when the parameter hardness 1 (hardness 1 in transgender cohort 22.12 ± 13.44 N compared to hardness 1 in oncological group 23.66 ± 13.54 N, *p* = 0.757; and hardness 2 (hardness 2 in transgender cohort 14.61 ± 10.31 N compared to hardness 2 in oncological group 15.30 ± 10.42 N, *p* = 0.856), cohesiveness (transgender cohesiveness 0.33 ± 0.05 compared to oncological cohesiveness 0.34 ± 0.08, *p* = 0.626) and springiness (transgender springiness 0.24 ± 0.05 N/mm compared to oncological stiffness 0.27 ± 0.14 N/mm, *p* = 0.283) were compared between transgender and oncological ovarian cortex. An incomplete recovery postcompression in both transgender and oncological patients resulted in a cohesiveness of 0.33 ± 0.05 and 0.34 ± 0.08 respectively and a springiness of 0.24 ± 0.05 and 0.27 ± 0.14 respectively clearly showing that the initial area under the curve (or area of work) is 3 times bigger than the second and that only a fourth of the initial peak force was needed during the second compression in both groups.

**Fig. 3:**
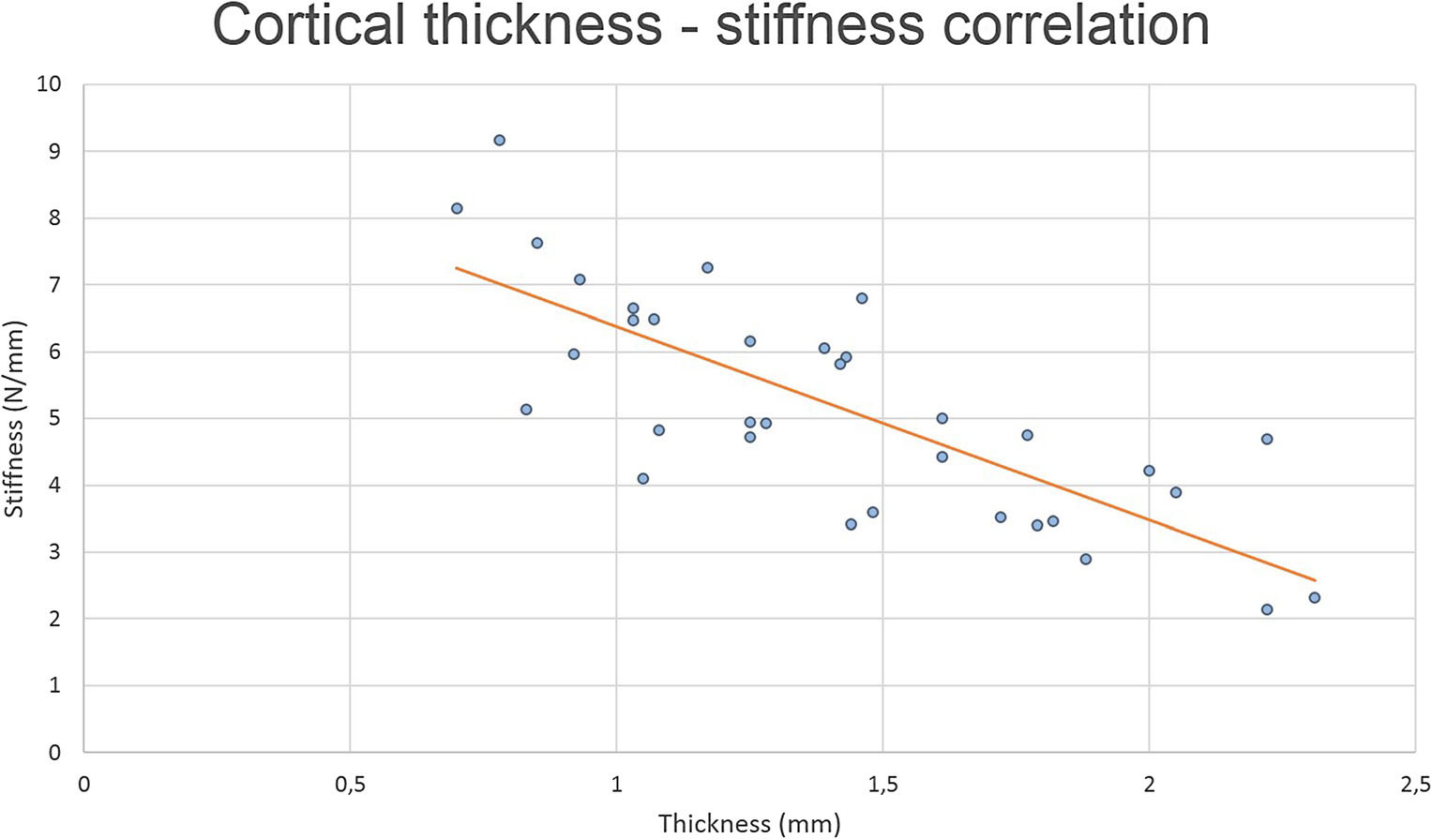
Cortical thickness and stiffness correlation**.** The tissue thickness correlated significantly with the stiffness (Rs − 0.785, *p* < 0.001)

In ovarian cortex derived from transgender men, tissue thickness correlated significantly with both the stiffness (Rs − 0.897, *p* < 0.001; shown in Fig. 3) and hardness parameters (hardness 1, Rs 0.483, *p* = 0.020; and hardness 2, Rs 0.535, *p* = 0.009). Hardness (1 and 2) also correlated significantly with the texture cohesiveness (Rs 0.429, *p* = 0.041; and Rs 0.565, *p* = 0.005 respectively). Also, springiness and cohesiveness were linearly correlated (Rs 0.470, *p* = 0.024).

The oncological tissue thickness correlated significantly with hardness 1 (Rs 0.615, *p* = 0.044). Again, springiness and cohesiveness were linearly correlated (Rs 0.845, *p* < 0.001).

**Fig. 4:**
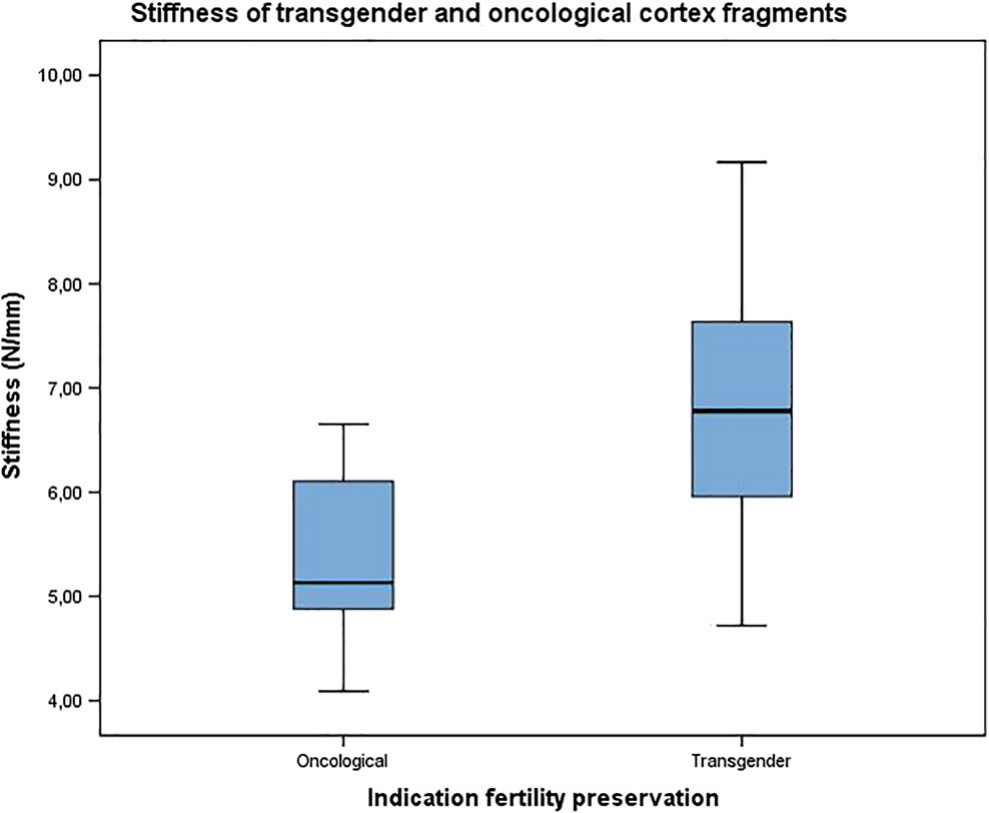
Stiffness of transgender men’s and oncological patient’s cortex fragments. Subanalysis of fragments < 1.4 mm (*N* transgender = 10 and *N* oncological = 7) showed a significantly different stiffness between transgender and oncological cortex fragments (*p* = 0.036)

Since thicker cortex fragments were negatively correlated with the lower degree of stiffness (overall Rs − 0.785, *p* < 0.001) as shown in Fig. 3, it could be possible that residual medulla on the cortex pieces influenced the measurements. Medulla tissue is softer than the cortex tissue and although preparation of the ovarian tissue for cryopreservation is performed by completely dissecting the medulla from the cortex, leaving a cortex thickness of 1–2 mm; it could be that residual medulla was present in the pieces as there is no clear exact macroscopic dissection plane between cortex and medulla. Cortical thickness has been reported to be between 1 and 2 mm, although detailed histological reports mention a cortex thickness of 1.5 mm (ranging from 1.4 to 1.6 mm) [9]. Taking into account this potential masking effect of the residual medulla on the result of the stiffness analyses, a subanalysis on the tissue fragments < 1.4 mm was performed. This selection included in 10 tissue fragments of transgender men and 7 pieces originating from oncological patients. Intriguingly, this resulted in a significantly different stiffness between cortex fragments of transgender and oncological patients (transgender stiffness 6.78 ± 1.38 N/mm compared to oncological stiffness 5.41 ± 0.9 N/mm, *p* = 0.036) (Fig. 4). The other parameters, being thickness, hardness 1 and 2, cohesiveness, and springiness, did not differ significantly between the 2 groups.

### Microscopic analysis of ovarian stromal tightness

**Fig. 5:**
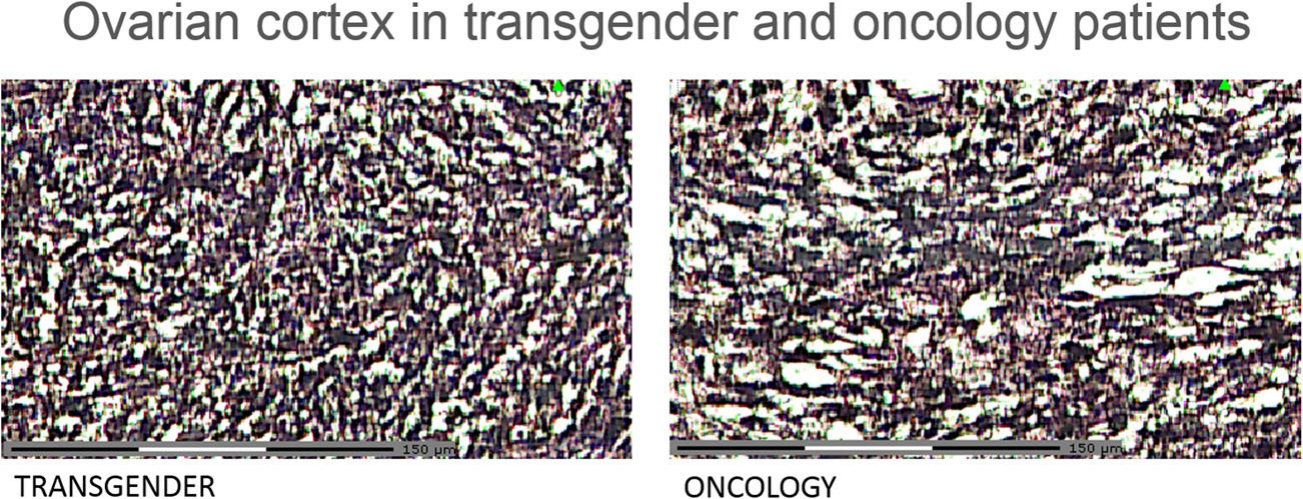
Ovarian cortex in transgender men and oncology patients: histological analysis (hematoxylin/eosin staining). Total scale bar = 150 μm

**Fig. 6:**
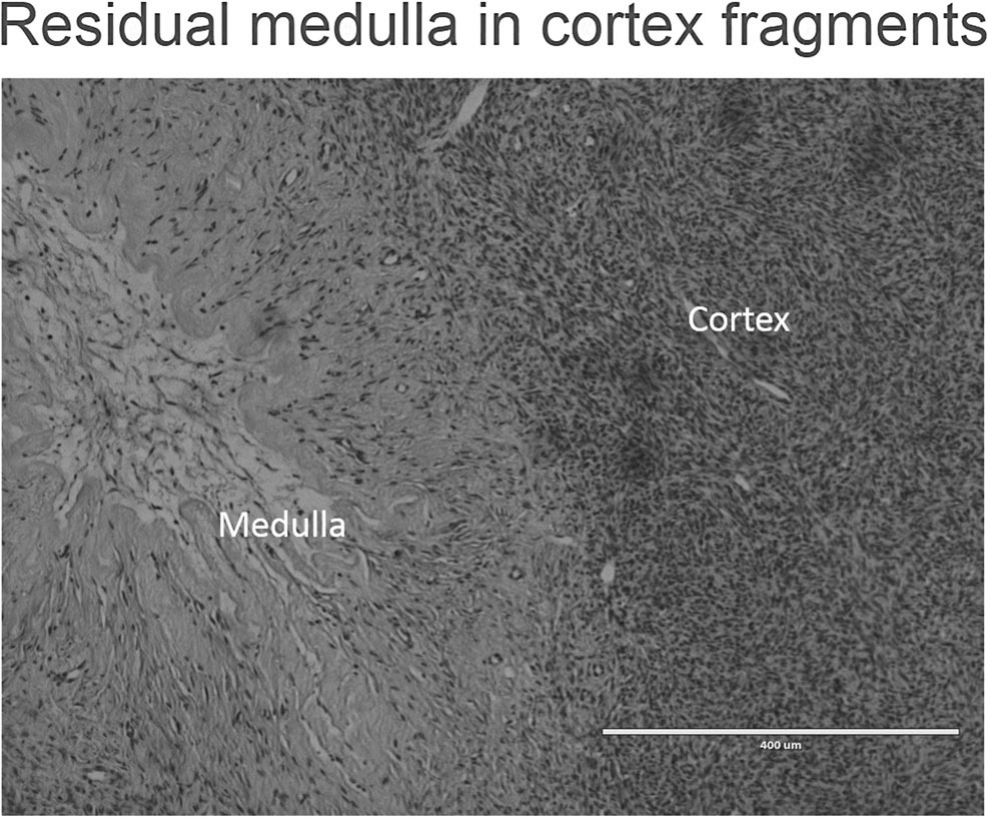
Residual medulla in cortex fragments (hematoxylin/eosin staining). Histological analysis also confirmed the presence of residual medulla after cortex preparation for cryopreservation. Scale bar = 400 μm

Figure 5 shows representative pictures of the ovarian stroma in oncology patients and transgender men. Histologic analysis also supports the idea of residual medulla after cortex preparation for cryopreservation, due to the absence of a clear macroscopic dissection plane between cortex and medulla (Fig. 6).

## Discussion and conclusion

This is the first application of TPA in ovarian cortex for measurement of textural ovarian cortical properties. Comparing the physical properties, we objectively describe an increased cortical stiffness in the most superficial part of the ovarian cortex following prolonged testosterone administration in transgender men compared to the non-exposed ovarian cortex of oncological patients.

TPA is a sensitive technique to measure textural properties, including stiffness, hardness, cohesiveness, and springiness [12] and is a preliminary and novel approach in ovarian research. Double compression of the ovarian fragments resulted in a first TPA curve with an increasing steepness and a prompt return in the first curve, followed by a smaller second curve. Processing the first results, a potential masking effect of the medulla on the stiffness results in the cortex was suspected as thinner fragments appeared to be significantly stiffer. Histological analysis confirmed the presence of residual medulla; however, we could not retrospectively measure the medullar thickness of the fragments used for TPA as these fragments were cut parallel to the cortical surface and a correct measurement of the medullar residue required a perpendicular cutting plane. Based on literature cutoffs, a strict subanalysis on the fragments < 1.4 mm was performed, assuming that the medulla was completely removed in these fragments. This subgroup analysis revealed a significantly different stiffness between the cortexes of transgender patients in comparison to cortex fragments from the oncological cohort. This confirms altered physical properties of the ovarian cortex, most possibly due to testosterone administration, at least in the most outer part of the cortex. This is in line with previous work, describing an increased subepithelial collagenization and stromal hyperplasia in the ovarian cortex after testosterone administration [9]. The altered stiffness particularly in the most superficial part of the cortex can be explained by the presence of androgen receptors in the ovarian surface epithelium [13]. Exposure of this epithelium to androgens results in DNA synthesis and in some cases a protection from cell death, leading to cell proliferation [14].

In this perspective, it could be of interest to repeat TPA on PCOS cortices to study the dimension of rigidity, hypothesizing that PCOS ovaries rigidity might constitute the intermediate state between ovaries in oncological patients (with normal levels of testosterone) and transgender men with intended supraphysiologic levels of testosterone as a result of the cross-sex hormone treatment. Some groups have even proposed to measure structural aspects of the ovarian cortex *in vivo*, previously described by Woodruff and Shea [15]. This group suggested and even tested multi-modal magnetic resonance elastography for *in vivo* assessment of the ovarian tissue rigidity [16].

A limitation of this work is the small sample size, inherent to this study subject. Cryopreserved tissue for fertility preservation is only scarcely available for research, especially if a large amount of tissue is needed to reduce inter patient variation as the variation in physical properties of cortex fragments is unknown thus far. Another weakness of this work is the inability to have an accurate measurement of the follicle density in the cortical fragments used for TPA, as the follicle number might influence the textural parameters of the ovarian cortex. Being processed for TPA, these fragments were no longer usable for other analyses. However, previous work revealed no quantitative difference in follicle counts and distributions comparing ovarian cortex derived from transgender men and oncological patients [17].

Additionally and more interestingly, TPA results can be directly translated to research in artificial ovary engineering. TPA is also applied in biomaterial industry and there is huge interest in the design of a suitable environment for *in vitro* follicle culture [2]. Especially for primordial follicles, attempts of *in vitro* culture appear to be more successful in tissue context as well as in artificial ovarian structures, if the 3D follicle-matrix structure is respected [18–20]. TPA parameters of the ovarian cortices described in our study can directly be set as a reference for optimization of the artificial matrix rigidity. In this respect, we recommend an overall matrix stiffness of 5.4 N/mm per 1 mm as a start rigidity. The TPA technique is also applicable to study and compare the physical parameters of these bioengineered matrices.

Not only research in designing artificial follicle maturation environments by using biomaterials but also the *in vitro* tissue follicle maturation research can benefit from studying TPA parameters in the ovarian cortex. Disrupting the follicle–matrix environment *in vitro* during tissue preparation for culture has proven to support follicle growth. The molecular basis of this mechanotransduction (the conversion of mechanical stimuli into biological responses) has been linked to the Hippo pathway signaling in ovarian physiology [21]. The Hippo pathway maintains the balance between negative growth regulators controlled by intact G-actin, whereas disruption of this pathway has shown to induce and support follicle development [21, 22]. Hippo pathway disruption is obtained by fragmentation of ovarian cortex during tissue preparation for *in vitro* culture [4, 21, 23, 24]. Since we have shown a difference in the cortical rigidity in the most outer part of the cortex after testosterone treatment, one may consider an adapted tissue preparation technique for Hippo pathway disruption according to the measured rigidity of the cortex fragment for culture.

In conclusion, this is the first application of TPA in ovarian cortex for measurement of textural ovarian cortical properties. Comparing the physical properties, we objectively describe an increased cortical stiffness in the most outer part of the ovarian cortex following prolonged testosterone administration in transgender men compared to the ovarian cortex of oncological patients. This preliminary and novel approach could be the start of future research to understand the physical properties of ovarian tissue.
